# Supplementary material for: Comparison of antibiotic use and antibiotic resistance between a community hospital and tertiary care hospital for evaluation of the antimicrobial stewardship program in Japan
Source: PLoS One. 2023 Apr 24;18(4):e0284806. doi: 10.1371/journal.pone.0284806 (PMC10124824; doi:10.1371/journal.pone.0284806)
Supplement: S1 Table — (PPTX) [file pone.0284806.s001.pptx]

## Slide 1
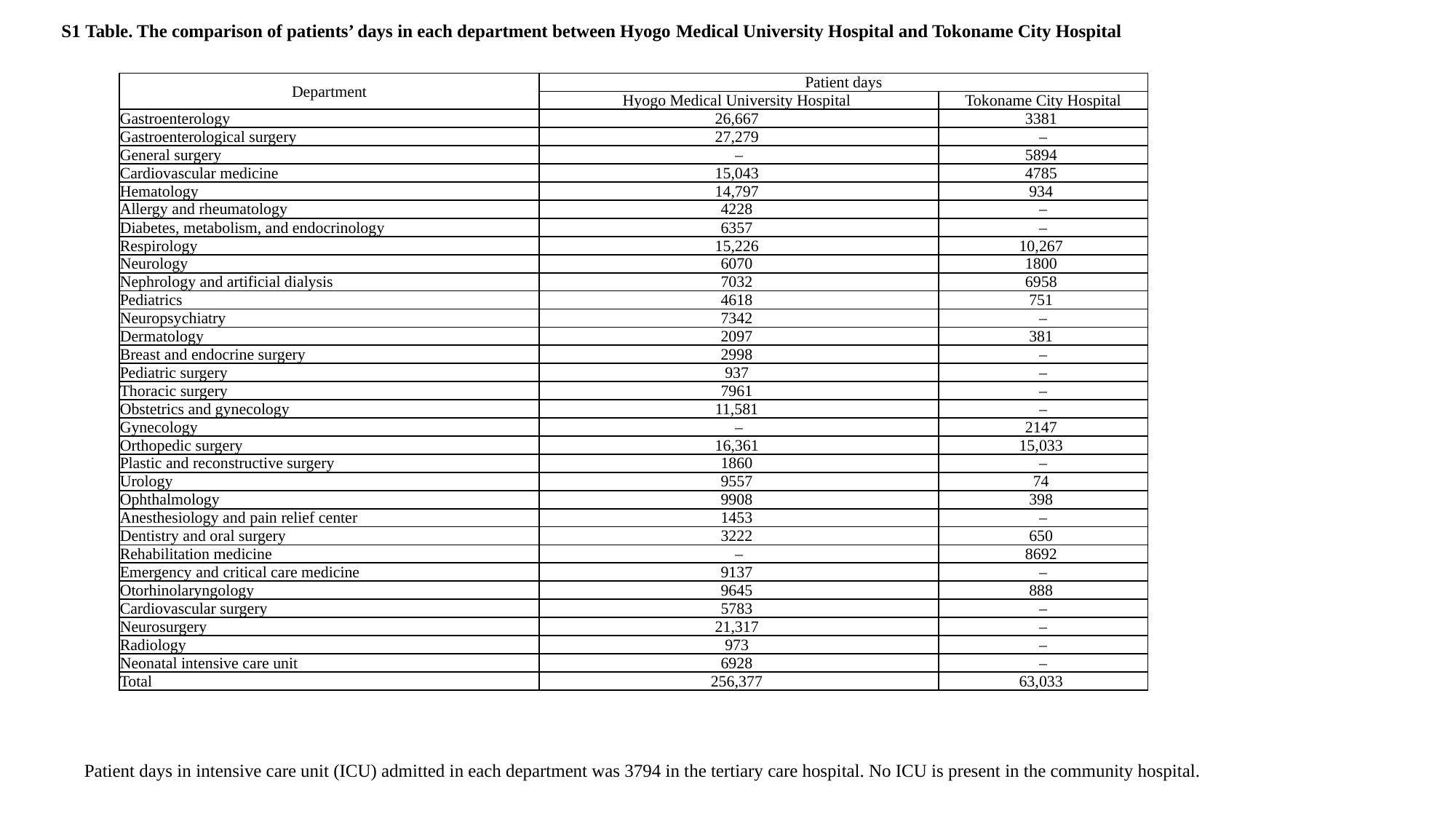

# S1 Table. The comparison of patients’ days in each department between Hyogo Medical University Hospital and Tokoname City Hospital
| Department | Patient days | |
| --- | --- | --- |
| | Hyogo Medical University Hospital | Tokoname City Hospital |
| Gastroenterology | 26,667 | 3381 |
| Gastroenterological surgery | 27,279 | – |
| General surgery | – | 5894 |
| Cardiovascular medicine | 15,043 | 4785 |
| Hematology | 14,797 | 934 |
| Allergy and rheumatology | 4228 | – |
| Diabetes, metabolism, and endocrinology | 6357 | – |
| Respirology | 15,226 | 10,267 |
| Neurology | 6070 | 1800 |
| Nephrology and artificial dialysis | 7032 | 6958 |
| Pediatrics | 4618 | 751 |
| Neuropsychiatry | 7342 | – |
| Dermatology | 2097 | 381 |
| Breast and endocrine surgery | 2998 | – |
| Pediatric surgery | 937 | – |
| Thoracic surgery | 7961 | – |
| Obstetrics and gynecology | 11,581 | – |
| Gynecology | – | 2147 |
| Orthopedic surgery | 16,361 | 15,033 |
| Plastic and reconstructive surgery | 1860 | – |
| Urology | 9557 | 74 |
| Ophthalmology | 9908 | 398 |
| Anesthesiology and pain relief center | 1453 | – |
| Dentistry and oral surgery | 3222 | 650 |
| Rehabilitation medicine | – | 8692 |
| Emergency and critical care medicine | 9137 | – |
| Otorhinolaryngology | 9645 | 888 |
| Cardiovascular surgery | 5783 | – |
| Neurosurgery | 21,317 | – |
| Radiology | 973 | – |
| Neonatal intensive care unit | 6928 | – |
| Total | 256,377 | 63,033 |
Patient days in intensive care unit (ICU) admitted in each department was 3794 in the tertiary care hospital. No ICU is present in the community hospital.
