# Supplementary material for: Comparison of antibiotic use and antibiotic resistance between a community hospital and tertiary care hospital for evaluation of the antimicrobial stewardship program in Japan
Source: PLoS One. 2023 Apr 24;18(4):e0284806. doi: 10.1371/journal.pone.0284806 (PMC10124824; doi:10.1371/journal.pone.0284806)
Supplement: S2 Table — (PPTX) [file pone.0284806.s002.pptx]

## Slide 1
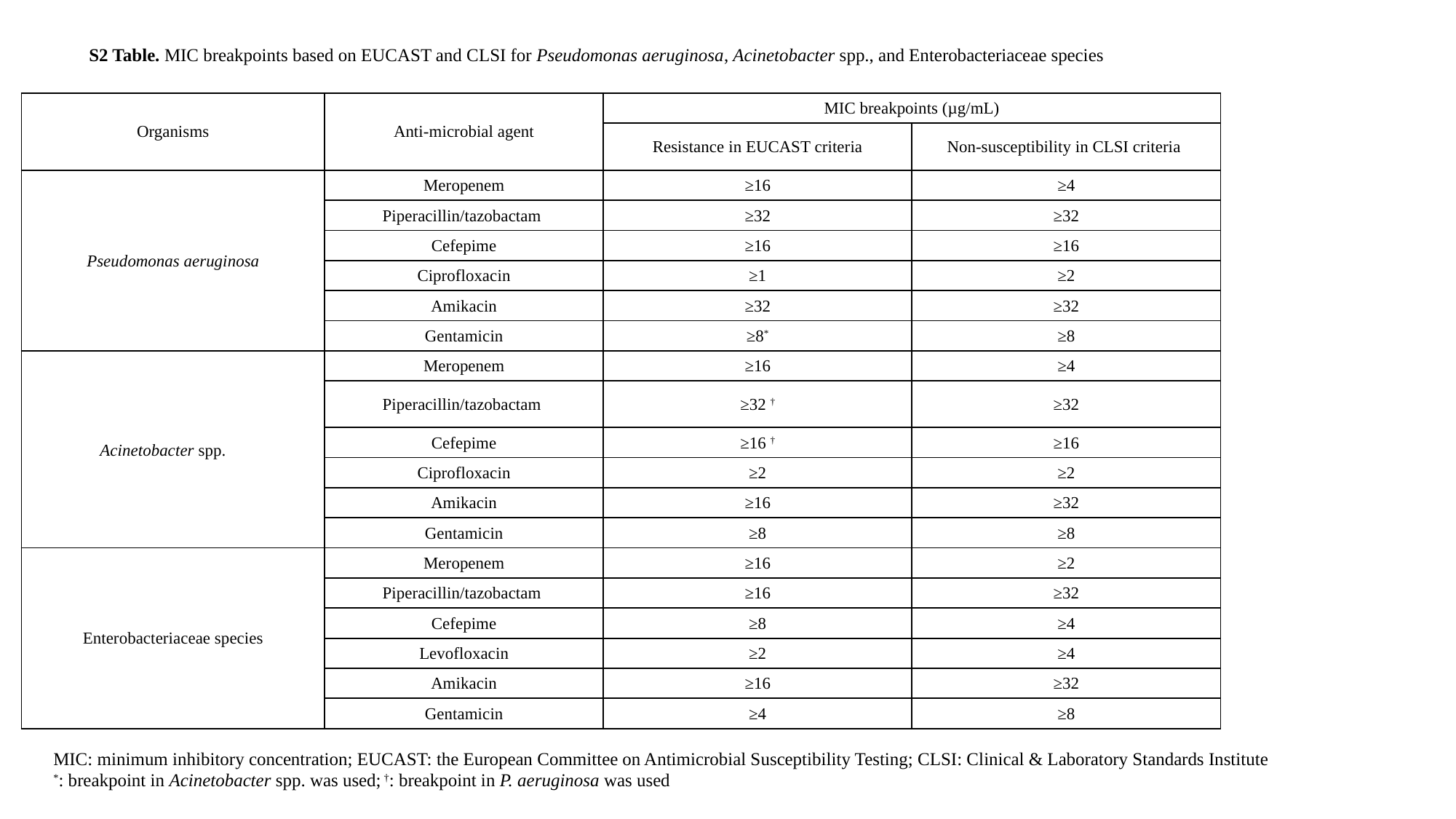

# S2 Table. MIC breakpoints based on EUCAST and CLSI for Pseudomonas aeruginosa, Acinetobacter spp., and Enterobacteriaceae species
| Organisms | Anti-microbial agent | MIC breakpoints (µg/mL) | |
| --- | --- | --- | --- |
| Organisms | 抗菌薬 | Resistance in EUCAST criteria | Non-susceptibility in CLSI criteria |
| Pseudomonas aeruginosa | Meropenem | ≥16 | ≥4 |
| | Piperacillin/tazobactam | ≥32 | ≥32 |
| | Cefepime | ≥16 | ≥16 |
| | Ciprofloxacin | ≥1 | ≥2 |
| | Amikacin | ≥32 | ≥32 |
| | Gentamicin | ≥8\* | ≥8 |
| Acinetobacter spp. | Meropenem | ≥16 | ≥4 |
| | Piperacillin/tazobactam | ≥32 † | ≥32 |
| | Cefepime | ≥16 † | ≥16 |
| | Ciprofloxacin | ≥2 | ≥2 |
| | Amikacin | ≥16 | ≥32 |
| | Gentamicin | ≥8 | ≥8 |
| Enterobacteriaceae species | Meropenem | ≥16 | ≥2 |
| | Piperacillin/tazobactam | ≥16 | ≥32 |
| | Cefepime | ≥8 | ≥4 |
| | Levofloxacin | ≥2 | ≥4 |
| | Amikacin | ≥16 | ≥32 |
| | Gentamicin | ≥4 | ≥8 |
MIC: minimum inhibitory concentration; EUCAST: the European Committee on Antimicrobial Susceptibility Testing; CLSI: Clinical & Laboratory Standards Institute
*: breakpoint in Acinetobacter spp. was used; †: breakpoint in P. aeruginosa was used
