# Supplementary material for: Comparison of antibiotic use and antibiotic resistance between a community hospital and tertiary care hospital for evaluation of the antimicrobial stewardship program in Japan
Source: PLoS One. 2023 Apr 24;18(4):e0284806. doi: 10.1371/journal.pone.0284806 (PMC10124824; doi:10.1371/journal.pone.0284806)
Supplement: S3 Table — (PPTX) [file pone.0284806.s003.pptx]

## Slide 1
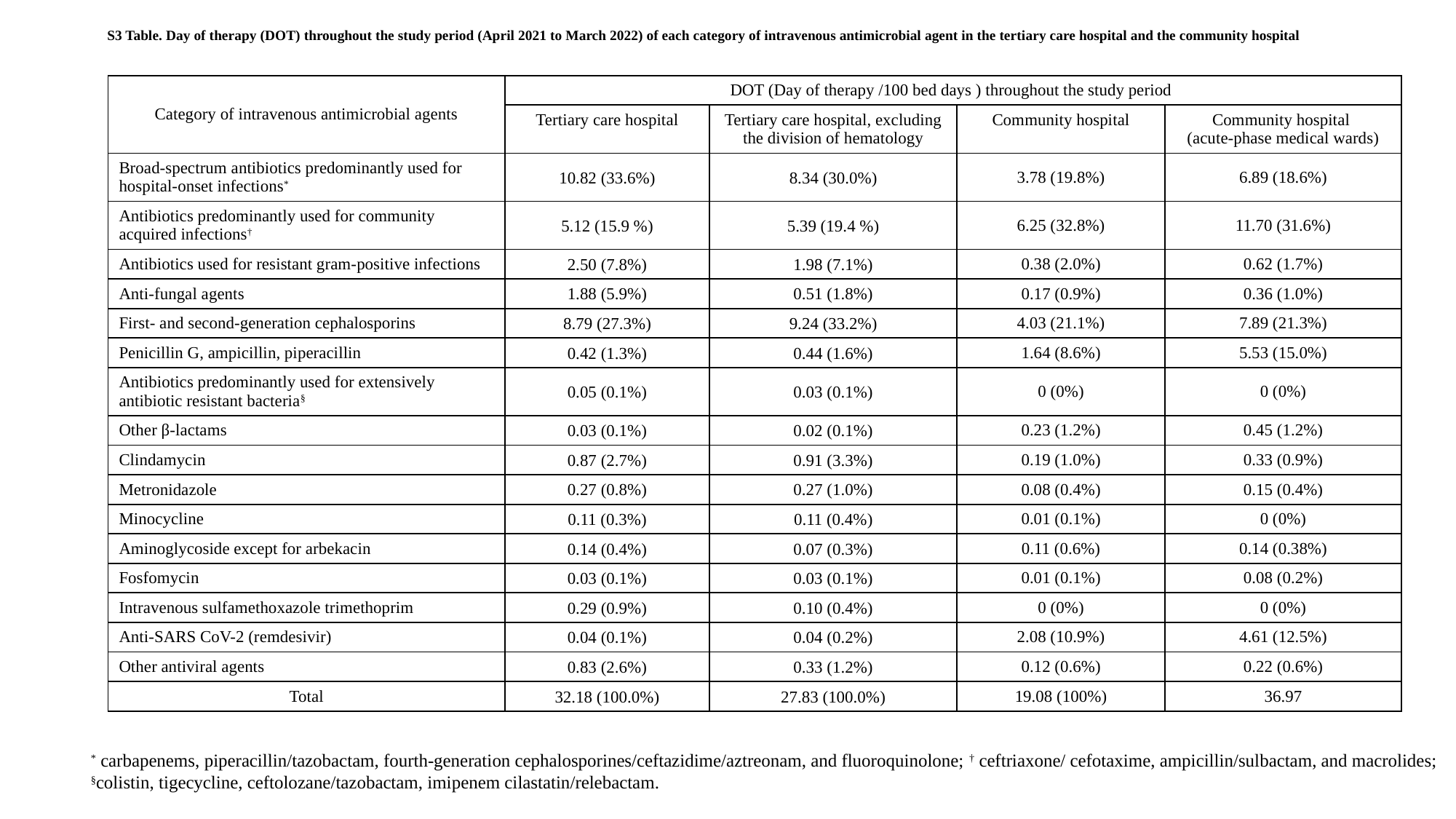

# S3 Table. Day of therapy (DOT) throughout the study period (April 2021 to March 2022) of each category of intravenous antimicrobial agent in the tertiary care hospital and the community hospital
| Category of intravenous antimicrobial agents | DOT (Day of therapy /100 bed days ) throughout the study period | | | |
| --- | --- | --- | --- | --- |
| | Tertiary care hospital | Tertiary care hospital, excluding the division of hematology | Community hospital | Community hospital (acute-phase medical wards) |
| Broad-spectrum antibiotics predominantly used for hospital-onset infections\* | 10.82 (33.6%) | 8.34 (30.0%) | 3.78 (19.8%) | 6.89 (18.6%) |
| Antibiotics predominantly used for community acquired infections† | 5.12 (15.9 %) | 5.39 (19.4 %) | 6.25 (32.8%) | 11.70 (31.6%) |
| Antibiotics used for resistant gram-positive infections | 2.50 (7.8%) | 1.98 (7.1%) | 0.38 (2.0%) | 0.62 (1.7%) |
| Anti-fungal agents | 1.88 (5.9%) | 0.51 (1.8%) | 0.17 (0.9%) | 0.36 (1.0%) |
| First- and second-generation cephalosporins | 8.79 (27.3%) | 9.24 (33.2%) | 4.03 (21.1%) | 7.89 (21.3%) |
| Penicillin G, ampicillin, piperacillin | 0.42 (1.3%) | 0.44 (1.6%) | 1.64 (8.6%) | 5.53 (15.0%) |
| Antibiotics predominantly used for extensively antibiotic resistant bacteria§ | 0.05 (0.1%) | 0.03 (0.1%) | 0 (0%) | 0 (0%) |
| Other β-lactams | 0.03 (0.1%) | 0.02 (0.1%) | 0.23 (1.2%) | 0.45 (1.2%) |
| Clindamycin | 0.87 (2.7%) | 0.91 (3.3%) | 0.19 (1.0%) | 0.33 (0.9%) |
| Metronidazole | 0.27 (0.8%) | 0.27 (1.0%) | 0.08 (0.4%) | 0.15 (0.4%) |
| Minocycline | 0.11 (0.3%) | 0.11 (0.4%) | 0.01 (0.1%) | 0 (0%) |
| Aminoglycoside except for arbekacin | 0.14 (0.4%) | 0.07 (0.3%) | 0.11 (0.6%) | 0.14 (0.38%) |
| Fosfomycin | 0.03 (0.1%) | 0.03 (0.1%) | 0.01 (0.1%) | 0.08 (0.2%) |
| Intravenous sulfamethoxazole trimethoprim | 0.29 (0.9%) | 0.10 (0.4%) | 0 (0%) | 0 (0%) |
| Anti-SARS CoV-2 (remdesivir) | 0.04 (0.1%) | 0.04 (0.2%) | 2.08 (10.9%) | 4.61 (12.5%) |
| Other antiviral agents | 0.83 (2.6%) | 0.33 (1.2%) | 0.12 (0.6%) | 0.22 (0.6%) |
| Total | 32.18 (100.0%) | 27.83 (100.0%) | 19.08 (100%) | 36.97 |
* carbapenems, piperacillin/tazobactam, fourth-generation cephalosporines/ceftazidime/aztreonam, and fluoroquinolone; † ceftriaxone/ cefotaxime, ampicillin/sulbactam, and macrolides; §colistin, tigecycline, ceftolozane/tazobactam, imipenem cilastatin/relebactam.
