# Supplementary material for: Comparison of antibiotic use and antibiotic resistance between a community hospital and tertiary care hospital for evaluation of the antimicrobial stewardship program in Japan
Source: PLoS One. 2023 Apr 24;18(4):e0284806. doi: 10.1371/journal.pone.0284806 (PMC10124824; doi:10.1371/journal.pone.0284806)
Supplement: S4 Table — (PPTX) [file pone.0284806.s004.pptx]

## Slide 1
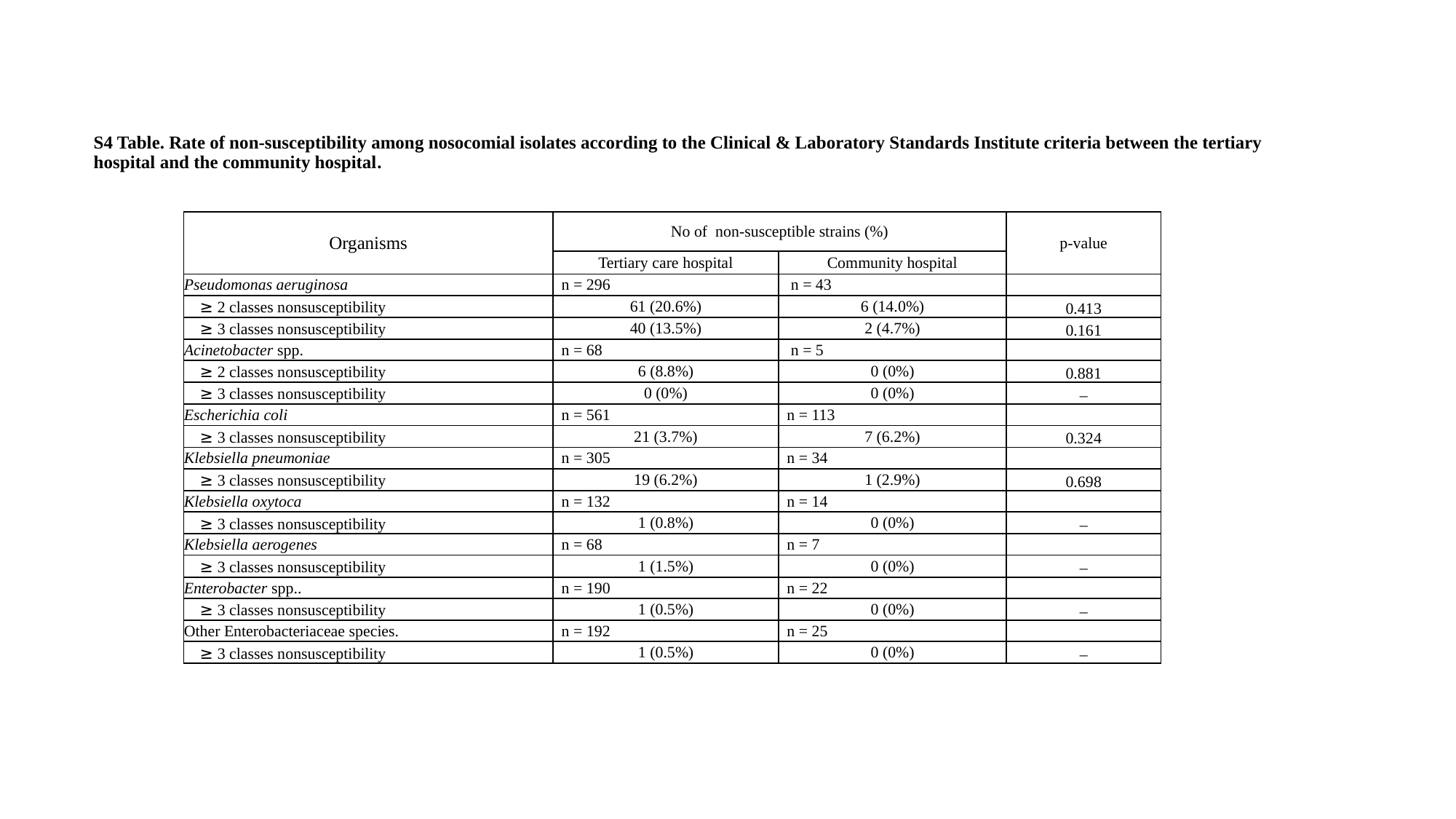

# S4 Table. Rate of non-susceptibility among nosocomial isolates according to the Clinical & Laboratory Standards Institute criteria between the tertiary hospital and the community hospital.
| Organisms | No of non-susceptible strains (%) | | p-value |
| --- | --- | --- | --- |
| | Tertiary care hospital | Community hospital | |
| Pseudomonas aeruginosa | n = 296 | n = 43 | |
| ≥2 classes nonsusceptibility | 61 (20.6%) | 6 (14.0%) | 0.413 |
| ≥3 classes nonsusceptibility | 40 (13.5%) | 2 (4.7%) | 0.161 |
| Acinetobacter spp. | n = 68 | n = 5 | |
| ≥2 classes nonsusceptibility | 6 (8.8%) | 0 (0%) | 0.881 |
| ≥3 classes nonsusceptibility | 0 (0%) | 0 (0%) | – |
| Escherichia coli | n = 561 | n = 113 | |
| ≥3 classes nonsusceptibility | 21 (3.7%) | 7 (6.2%) | 0.324 |
| Klebsiella pneumoniae | n = 305 | n = 34 | |
| ≥3 classes nonsusceptibility | 19 (6.2%) | 1 (2.9%) | 0.698 |
| Klebsiella oxytoca | n = 132 | n = 14 | |
| ≥3 classes nonsusceptibility | 1 (0.8%) | 0 (0%) | – |
| Klebsiella aerogenes | n = 68 | n = 7 | |
| ≥3 classes nonsusceptibility | 1 (1.5%) | 0 (0%) | – |
| Enterobacter spp.. | n = 190 | n = 22 | |
| ≥3 classes nonsusceptibility | 1 (0.5%) | 0 (0%) | – |
| Other Enterobacteriaceae species. | n = 192 | n = 25 | |
| ≥3 classes nonsusceptibility | 1 (0.5%) | 0 (0%) | – |
