# Supplementary material for: Comparison of antibiotic use and antibiotic resistance between a community hospital and tertiary care hospital for evaluation of the antimicrobial stewardship program in Japan
Source: PLoS One. 2023 Apr 24;18(4):e0284806. doi: 10.1371/journal.pone.0284806 (PMC10124824; doi:10.1371/journal.pone.0284806)
Supplement: S5 Table — (PPTX) [file pone.0284806.s005.pptx]

## Slide 1
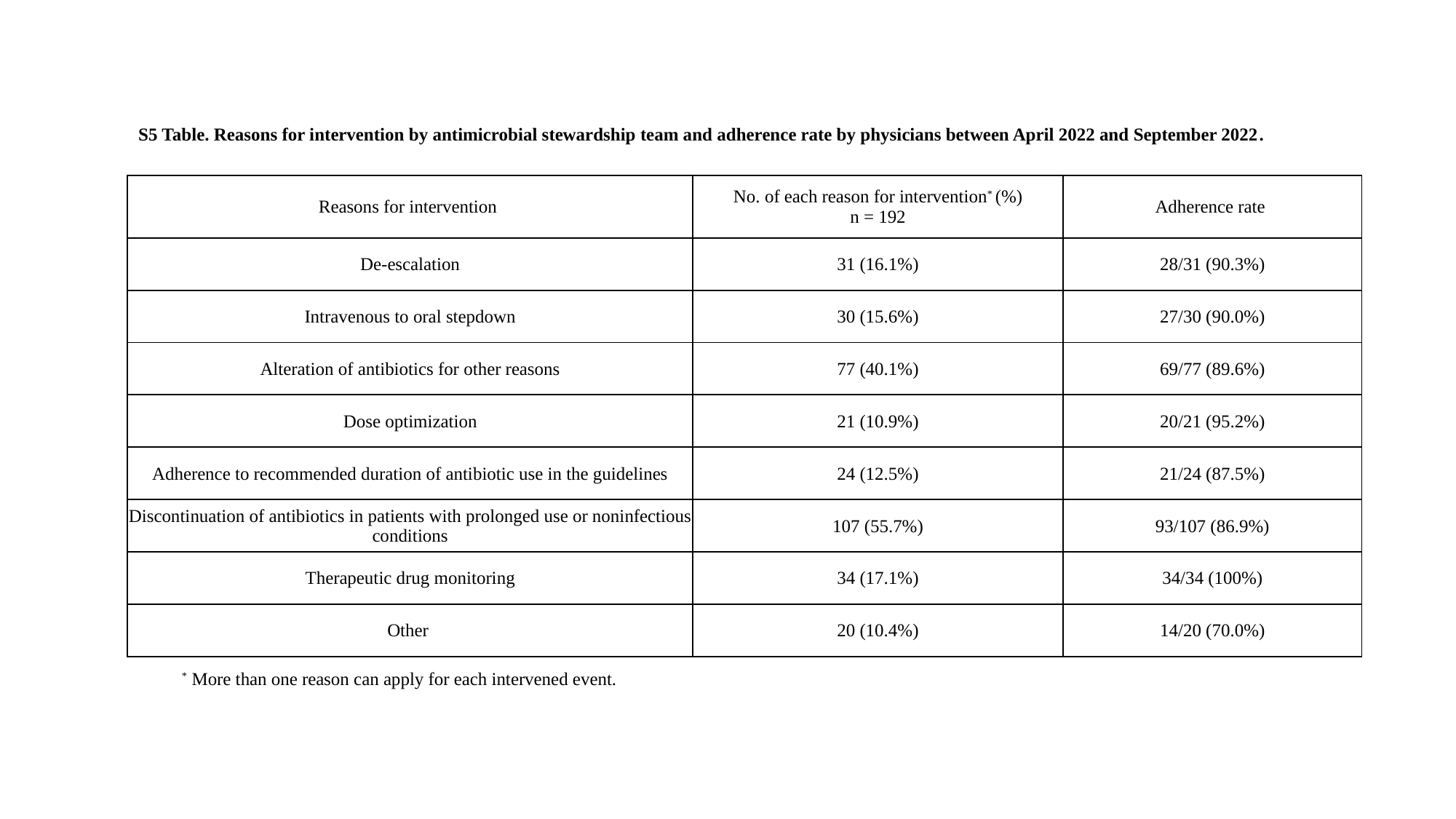

# S5 Table. Reasons for intervention by antimicrobial stewardship team and adherence rate by physicians between April 2022 and September 2022.
| Reasons for intervention | No. of each reason for intervention\* (%) n = 192 | Adherence rate |
| --- | --- | --- |
| De-escalation | 31 (16.1%) | 28/31 (90.3%) |
| Intravenous to oral stepdown | 30 (15.6%) | 27/30 (90.0%) |
| Alteration of antibiotics for other reasons | 77 (40.1%) | 69/77 (89.6%) |
| Dose optimization | 21 (10.9%) | 20/21 (95.2%) |
| Adherence to recommended duration of antibiotic use in the guidelines | 24 (12.5%) | 21/24 (87.5%) |
| Discontinuation of antibiotics in patients with prolonged use or noninfectious conditions | 107 (55.7%) | 93/107 (86.9%) |
| Therapeutic drug monitoring | 34 (17.1%) | 34/34 (100%) |
| Other | 20 (10.4%) | 14/20 (70.0%) |
* More than one reason can apply for each intervened event.
