# Supplementary material for: Comparison of antibiotic use and antibiotic resistance between a community hospital and tertiary care hospital for evaluation of the antimicrobial stewardship program in Japan
Source: PLoS One. 2023 Apr 24;18(4):e0284806. doi: 10.1371/journal.pone.0284806 (PMC10124824; doi:10.1371/journal.pone.0284806)
Supplement: S6 Table — (PPTX) [file pone.0284806.s006.pptx]

## Slide 1
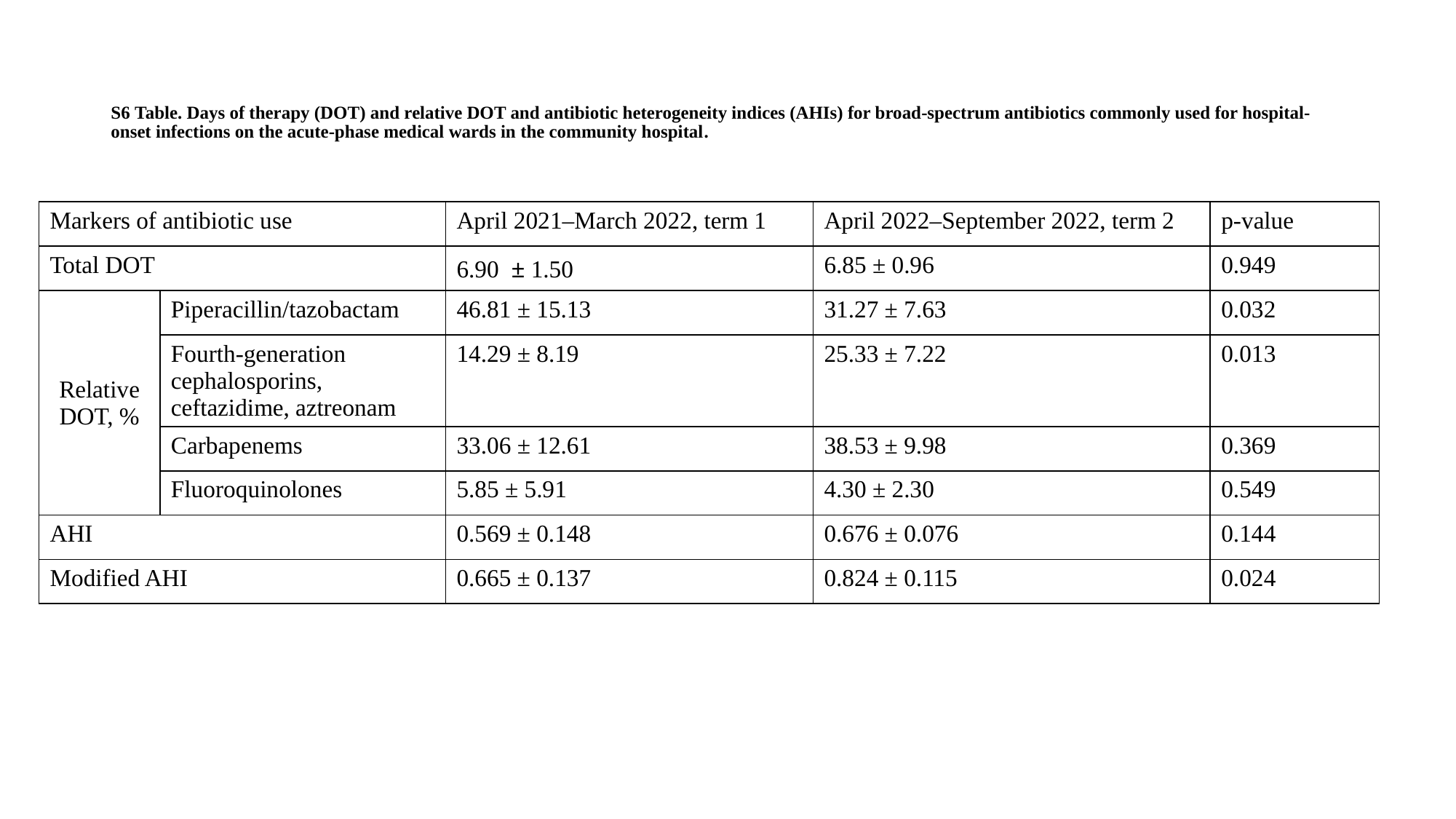

# S6 Table. Days of therapy (DOT) and relative DOT and antibiotic heterogeneity indices (AHIs) for broad-spectrum antibiotics commonly used for hospital-onset infections on the acute-phase medical wards in the community hospital.
| Markers of antibiotic use | | April 2021–March 2022, term 1 | April 2022–September 2022, term 2 | p-value |
| --- | --- | --- | --- | --- |
| Total DOT | | 6.90 ± 1.50 | 6.85 ± 0.96 | 0.949 |
| Relative DOT, % | Piperacillin/tazobactam | 46.81 ± 15.13 | 31.27 ± 7.63 | 0.032 |
| | Fourth-generation cephalosporins, ceftazidime, aztreonam | 14.29 ± 8.19 | 25.33 ± 7.22 | 0.013 |
| | Carbapenems | 33.06 ± 12.61 | 38.53 ± 9.98 | 0.369 |
| | Fluoroquinolones | 5.85 ± 5.91 | 4.30 ± 2.30 | 0.549 |
| AHI | | 0.569 ± 0.148 | 0.676 ± 0.076 | 0.144 |
| Modified AHI | | 0.665 ± 0.137 | 0.824 ± 0.115 | 0.024 |
