# Supplementary material for: Comparison of antibiotic use and antibiotic resistance between a community hospital and tertiary care hospital for evaluation of the antimicrobial stewardship program in Japan
Source: PLoS One. 2023 Apr 24;18(4):e0284806. doi: 10.1371/journal.pone.0284806 (PMC10124824; doi:10.1371/journal.pone.0284806)
Supplement: S1 Fig — (PPTX) [file pone.0284806.s007.pptx]

## Slide 1
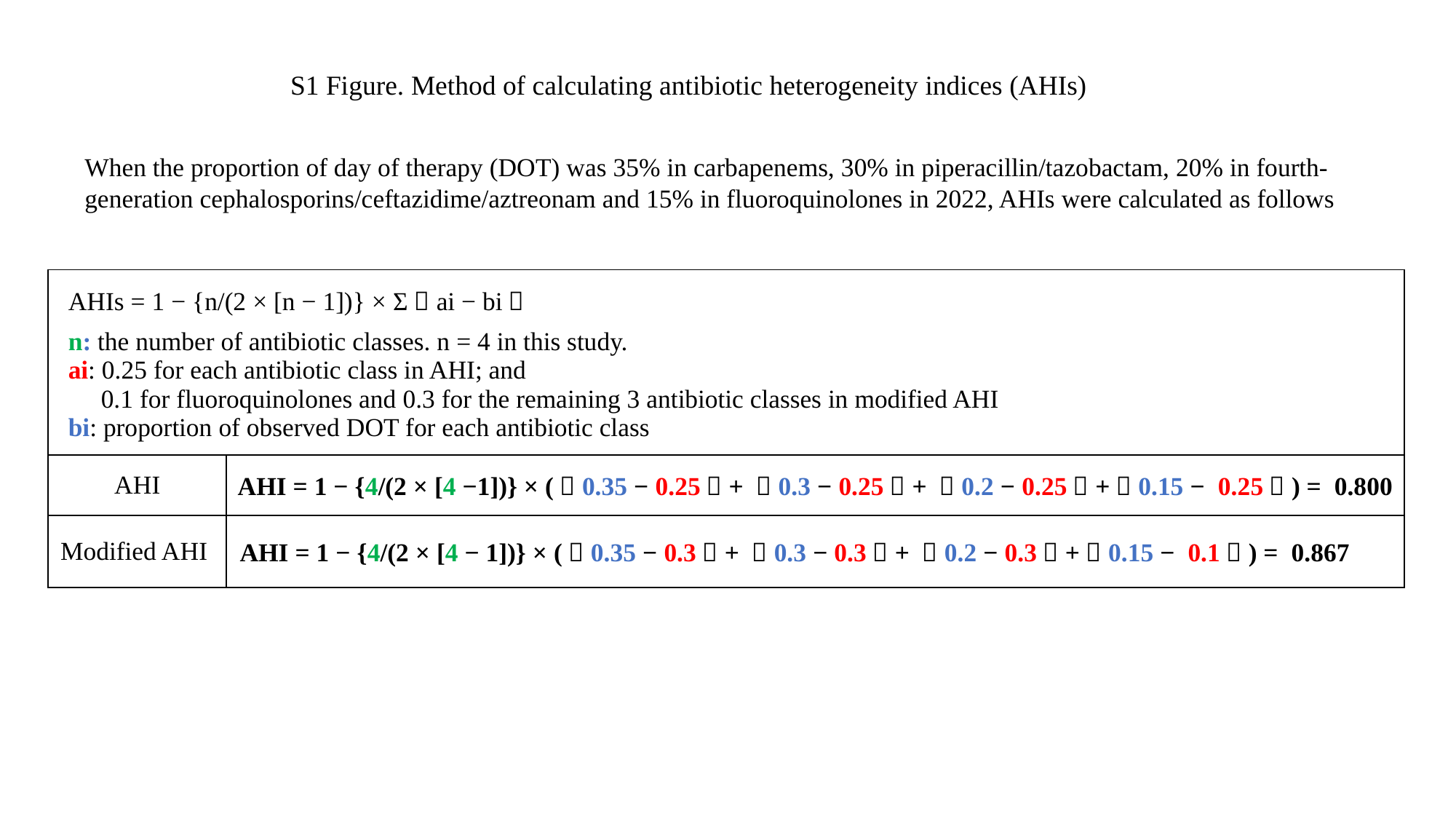

S1 Figure. Method of calculating antibiotic heterogeneity indices (AHIs)
When the proportion of day of therapy (DOT) was 35% in carbapenems, 30% in piperacillin/tazobactam, 20% in fourth-generation cephalosporins/ceftazidime/aztreonam and 15% in fluoroquinolones in 2022, AHIs were calculated as follows
| AHIs = 1 − {n/(2 × [n − 1])} × Σ｜ai − bi｜ n: the number of antibiotic classes. n = 4 in this study. ai: 0.25 for each antibiotic class in AHI; and 0.1 for fluoroquinolones and 0.3 for the remaining 3 antibiotic classes in modified AHI bi: proportion of observed DOT for each antibiotic class | |
| --- | --- |
| AHI | AHI = 1 − {4/(2 × [4 −1])} × (｜0.35 − 0.25｜+ ｜0.3 − 0.25｜+ ｜0.2 − 0.25｜+｜0.15 − 0.25｜) = 0.800 |
| Modified AHI | AHI = 1 − {4/(2 × [4 − 1])} × (｜0.35 − 0.3｜+ ｜0.3 − 0.3｜+ ｜0.2 − 0.3｜+｜0.15 − 0.1｜) = 0.867 |
